# Effectiveness of Low-Dose Atropine for Myopia Control in European Children: A Real-World Cohort Study

**DOI:** 10.3390/jcm15114393

**Published:** 2026-06-05

**Authors:** Cristina Alvarez-Peregrina, Paola Marcela Pinzón Martínez, Carlos Navarro Villanueva, Pablo De Gracia, Manuel Moriche-Carretero, Maria Teresa Cedazo-Anton, Raquel Coca-Serrano, Miguel Ángel Sánchez-Tena

**Affiliations:** 1 Department of Optometry and Vision, Faculty of Optics and Optometry, Universidad Complutense de Madrid, 28037 Madrid, Spain; cristina_alvarez@ucm.es (C.A.-P.); masancheztena@ucm.es (M.Á.S.-T.); 2 School of Optometry, University of Detroit Mercy, Novi, MI 48377, USA; 3 Hospital Universitario Infanta Sofía, 28702 Madrid, Spain; mmoriche@gmail.com (M.M.-C.);; 4 Faculty of Biomedical and Health Sciences, Universidad Europea de Madrid, 28670 Madrid, Spain; 5 ISEC LISBOA—Instituto Superior de Educação e Ciências, 1750-179 Lisbon, Portugal

**Keywords:** myopia, atropine, axial length, child, treatment outcome

## Abstract

**Purpose**: To evaluate the real-world effectiveness of low-dose atropine in slowing myopia progression in European children and to contextualize axial elongation using an age- and race-adjusted epidemiological model. **Methods**: This retrospective, longitudinal cohort study included 76 children (mean age 8.7 ± 1.5 years) with myopia treated with atropine 0.01%, 0.025%, or 0.05% at a tertiary hospital in Madrid. Cycloplegic spherical equivalent (SE) and axial length (AL) were recorded at baseline and 12 months. Expected untreated AL elongation was estimated using a published meta-regression model. Observed and expected AL changes were compared using paired *t*-tests. Predictors of SE and AL progression were assessed using multiple linear regression including age, sex, baseline SE or AL, atropine concentration, adherence, ethnicity, and family history. **Results**: Mean SE progression was −0.08 ± 0.11 D per year, and mean AL elongation was 0.08 ± 0.23 mm per year. Expected untreated elongation was 0.36 ± 0.09 mm per year and significantly exceeded the observed elongation (*p* < 0.001), representing a 78% relative reduction (effect size Cohen’s d = −1.18). Older age (≥9 years) and more negative baseline SE were associated with greater SE progression (*p* < 0.05). Adverse effects and treatment tolerability were not systematically evaluated in this retrospective cohort. No significant differences were found across atropine concentrations, adherence levels, sex, ethnicity, or family history. The regression model for AL progression was not statistically significant. **Conclusions**: Low-dose atropine demonstrated real-world effectiveness in reducing myopia progression in European children. Axial elongation was markedly lower than epidemiological expectations, supporting low-dose atropine as a first-line therapy in routine clinical practice. Age and baseline refractive error were significant predictors of treatment response.

## 1. Introduction

Myopia is a refractive error in which light rays coming from a distant object focus in front of the retina, rather than directly on it, resulting in blurred distance vision. Once considered an optical inconvenience, myopia is now recognized as a global public health issue due to its increasing prevalence and associated sight-threatening complications. High myopia (typically defined as spherical equivalent (SE) ≤ −6.00 D or axial length (AL) ≥ 26 mm) has been strongly linked to retinal detachment, myopic maculopathy, open-angle glaucoma, and early-onset cataracts, with high personal and societal costs [1,2,3].

Epidemiological projections suggest that by 2050, nearly 50% of the global population will be myopic, and 10% will develop high myopia [2]. While genetics plays a role in susceptibility, the rapid increase in prevalence over the past few decades, points to strong environmental contributions, most notably decreased outdoor time and increased near-work activity, including screen use [4,5,6]. This behavioral shift was further exacerbated by the COVID-19 pandemic [7], which confinement led to reduced outdoor exposure and greater digital device reliance, especially in children.

Traditionally, the clinical approach to myopia focused on refractive correction rather than attempting to slow its progression. However, in recent years, there has been a shift toward active myopia control strategies, including optical interventions (e.g., orthokeratology, defocus-modifying lenses) and pharmacological approaches [8]. Among pharmacologic options, low-dose atropine eye drops (typically 0.01% to 0.05%) have emerged as one of the most effective and widely studied interventions. Landmark randomized clinical trials (RCTs) such as the ATOM (Singapore and India) and LAMP (Hong Kong) studies demonstrated significant reductions in refractive and axial progression in atropine-treated children compared to placebo [9,10,11,12,13,14,15,16,17]. Despite substantial evidence from RCTs, questions remain about the applicability of these findings in routine clinical practice. Clinical trials often include homogeneous populations, standardized protocols, and high-frequency follow-up, which may not reflect typical care settings. Adherence variability, differing prescribing patterns, and diverse patient demographics all impact treatment outcomes in daily practice [18].

Although low-dose atropine has become widely adopted in several East Asian countries following the ATOM and LAMP trials, its implementation in Europe has been comparatively slower and more heterogeneous. Differences in regulatory pathways, prescribing practices, access to compounded formulations, and clinician familiarity with pharmacologic myopia control have contributed to substantial variability in atropine use across European countries. Much of the current evidence base originates from Asian populations, where baseline myopia prevalence, progression rates, environmental exposures, and iris pigmentation may differ substantially from those observed in European children. Emerging European studies such as MOSAIC and recent Spanish cohorts have demonstrated encouraging results with low-dose atropine, but real-world data in Western pediatric populations remain limited compared with the extensive literature available from East and Southeast Asia. Consequently, additional European real-world studies are important to better characterize treatment effectiveness, tolerability, and clinical applicability in populations that may differ ethnically, environmentally, and behaviorally from those traditionally represented in randomized clinical trials. Furthermore, there is a growing need for studies that reflect the heterogeneity of routine clinical practice, including variability in adherence, prescribing patterns, and follow-up intervals. Conducting controlled studies with untreated comparison groups has become increasingly difficult and ethically questionable, given the well-established efficacy of atropine in slowing myopia progression. As a result, alternative approaches such as epidemiological benchmarks or model-based estimates of expected progression are essential to contextualize treatment effects in contemporary cohorts. In addition to evaluating the real-world effectiveness of low-dose atropine in a European pediatric population, this study compares observed axial elongation with age- and race-adjusted expectations derived from published meta-regression data.

Within this context, the choice of outcome measures becomes particularly important when interpreting treatment efficacy in retrospective real-world cohorts. Axial length has emerged as one of the most important outcome measures in contemporary myopia research because it provides a structural biomarker of ocular growth that is less influenced by short-term accommodative fluctuations than refractive error measurements alone. In addition, excessive axial elongation is directly associated with the long-term risk of myopic retinal complications, making it a clinically meaningful endpoint for evaluating treatment efficacy. As placebo-controlled trials become increasingly difficult to justify ethically in progressing myopic children, alternative analytical strategies are needed to contextualize treatment outcomes in real-world cohorts. Epidemiological benchmarking using age- and race-adjusted growth models represents one potential approach for estimating expected untreated progression while avoiding direct treatment withholding. Although these model-based approaches cannot fully replace contemporaneous control groups and remain dependent on the characteristics of the populations from which they were derived, they may provide clinically useful reference frameworks for interpreting treatment effects in retrospective or observational studies. The integration of epidemiological modeling with real-world clinical data may therefore become increasingly relevant in future myopia-control research, particularly in settings where randomized untreated controls are not feasible.

To bridge this gap, this retrospective study evaluated the effectiveness of low-dose atropine in a European pediatric population attending a public tertiary hospital in Madrid, Spain. In addition to assessing refractive and axial outcomes under routine clinical conditions, the study compared observed axial elongation with age- and race-adjusted epidemiological expectations derived from a published meta-regression model.

## 2. Materials/Subjects and Methods

### 2.1. Study Design

This was a retrospective, longitudinal, observational study designed to evaluate the real-world effectiveness of low-dose atropine eye drops in controlling myopia progression in children. The study was conducted using anonymized clinical records of pediatric patients treated at the Ophthalmology Department of the Hospital Universitario Infanta Sofía (Madrid, Spain) between January 2021 and December 2023. The study adhered to the principles of the Declaration of Helsinki and was approved by the Research Ethics Committee of Hospital Clínico San Carlos in November 2024 (code C.I. 24/739-O_M_NoSP).

### 2.2. Participants

Seventy-six children aged 5 to 16 years at the initiation of treatment who had been diagnosed with myopia, defined as a spherical equivalent of ≤−0.50 D, and who were prescribed atropine at concentrations of 0.01%, 0.025%, or 0.05% were eligible for inclusion. Participants were required to have at least two clinical visits with complete objective refraction data separated by a minimum of 12 months.

### 2.3. Exclusion Criteria

Patients were excluded from the study if they presented with ocular comorbidities such as amblyopia, strabismus, or retinal pathology; systemic conditions known to affect vision; incomplete medical records; or if they were undergoing concurrent treatment with other myopia control methods, including orthokeratology, soft contact lenses or spectacles designed for myopia control.

### 2.4. Data Collection

Data were collected retrospectively from electronic medical records and included the following variables: patient age and gender, cycloplegic refraction, and AL measured by the IOL Master 500 or 700 (Carl Zeiss Meditec, Jena, Germany). Cycloplegic refraction was performed as part of routine pediatric ophthalmic care using standard clinical protocols. Refractions were obtained after pharmacologic cycloplegia to minimize accommodative fluctuations and improve the accuracy of refractive measurements in children. Spherical equivalent was calculated as the spherical power plus half of the cylindrical correction. Axial length measurements were acquired using non-contact optical biometry with the IOLMaster 500 or IOLMaster 700, both of which are widely used and validated devices in myopia research and clinical practice. Whenever possible, the same biometry platform was used consistently across follow-up visits for each participant to minimize inter-device variability. All measurements were obtained during routine clinical examinations conducted by experienced ophthalmic personnel under standard pediatric clinical conditions.

Information regarding the prescribed atropine concentration and treatment adherence, categorized as high (≥75% compliance) or low (<75%) based on clinical notes and parental reports, was also recorded. Additional data included the duration of follow-up and any documented adverse effects. Only data from the right eye were analyzed, given the strong interocular correlation observed for SE (r = 0.91) and AL (r = 0.96).

### 2.5. Outcome Measures

The primary outcomes were annual progression of SE (diopters/year) and AL (mm/year). Secondary outcomes included treatment concentration, adherence, age, SE, and AL at treatment initiation, and the correlation between changes in SE and AL.

### 2.6. Expected Untreated Axial Elongation (Model-Based Benchmark)

To contextualize the observed axial elongation under atropine treatment, the expected axial elongation in the absence of atropine was estimated using the meta-regression model published by Brennan et al. [19], which quantifies the influence of age and race on axial growth in untreated myopic children.

The model predicts annual axial elongation (in mm/year) as:

ln(E[ΔAL])=0.362−0.158(Age+0.5)+0.325(RaceCode),

where:

- Age = age in years at treatment initiation;
- RaceCode = 0 for Caucasian and 1 for non-Caucasian ethnicity.

For each participant with available AL measurements at baseline and 12 months, the expected axial elongation was computed as:

E[ΔAL]=exp(ln(E[ΔAL])).

Observed axial elongation was calculated as:

$$\Delta AL_{\mathrm{obs}}=AL_{12m}-AL_{\mathrm{baseline}}.$$

Absolute and relative reductions in axial elongation were derived as:

$$\Delta AL_{\mathrm{diff}}=E[\Delta AL]-\Delta AL_{\mathrm{obs}},$$

$$\mathrm{Relative}\;\mathrm{reduction}=1-\frac{\Delta AL_{\mathrm{obs}}}{E[\Delta AL]}.$$

This model-based benchmark provides an epidemiological reference for expected progression in untreated children, avoiding the need for an ethically problematic untreated control group.

### 2.7. Statistical Analysis

A statistical analysis was conducted to evaluate the one-year progression of myopia, using changes in SE and AL as dependent variables. Independent samples *t*-tests were performed to compare SE and AL progression across categorical variables, including sex, age (dichotomized as ≥9 vs. <9 years), treatment adherence, atropine concentration, ethnicity, and family history of myopia. Pearson correlation coefficients were calculated to assess the relationship between atropine concentration and changes in SE and AL.

Multiple linear regression models were constructed to identify independent predictors of SE and AL progression. Explanatory variables included age, sex, atropine concentration, adherence, ethnicity, family history of myopia, and baseline SE or AL. Model assumptions were verified through inspection of residuals.

For the model-based comparison of observed versus expected axial elongation, expected AL change was calculated for each participant using the Brennan et al. meta-regression equation. Observed and expected axial elongation values were compared using paired *t*-tests. Absolute and relative reductions in axial elongation were derived from these values, and effect sizes were quantified using Cohen’s d (with Hedges’ correction for small sample bias). Statistical significance was set at *p* < 0.05. All analyses were performed using SPSS version 27 (IBM Corp., Armonk, NY, USA).

## 3. Results

### 3.1. Participant Characteristics

A total of 76 children (mean age: 8.7  ±  1.5 years; 56.6% female) met the inclusion criteria. All were diagnosed with moderate myopia and treated with atropine eye drops at concentrations of 0.01%, 0.025%, or 0.05%. Baseline mean SE was −2.52  ±  0.80 D, and AL was 24.42  ±  0.99 mm. SE data were available for all 76 participants, while AL measurements were only available for 36 of them. Table 1 shows the baseline data of the participants.

### 3.2. Myopia Progression and Axial Elongation

After one year of treatment, mean SE progression in the right eye was −0.08 ± 0.11 D, with a median of −0.10 D (IQR: −0.13 to 0.00 D). Mean axial elongation was 0.08 ± 0.23 mm, with a median of 0.05 mm (IQR: −0.01 to 0.10 mm). Table 2 presents SE and AL changes after one year.

### 3.3. Subgroup Analyses

No statistically significant differences in SE or AL progression were found according to sex, treatment adherence, atropine concentration, or family history of myopia (all *p* > 0.05).

Age at treatment initiation showed a significant effect on SE progression. Children aged ≥9 years exhibited greater myopic progression (−0.11 ± 0.11 D) compared with those <9 years (−0.04 ± 0.11 D; *p* = 0.007, Cohen’s d = −0.60). No significant differences in AL progression were observed between age groups.

When stratified by atropine concentration, SE progression was -0.09 ± 0.11 D in the 0.01% group, −0.09 ± 0.13 D in the 0.025% group, and −0.05 ± 0.08 D in the 0.05% group, with no statistically significant differences (*p* = 0.36).

**Figure 1:**
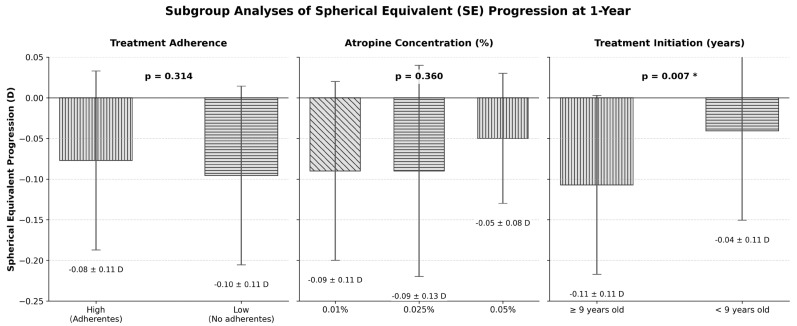
Subgroup analysis of spherical equivalent (SE) progression under atropine treatment. * Three bar plots summarise SE progression (in dioptres) across key subgroups, with each bar representing the mean ± standard deviation. (**Left**) Comparison by treatment adherence: no significant differences between children with high (≥75%) and low adherence. (**Centre**) Comparison by atropine concentration: No significant differences among different atropine concentrations were observed. (**Right**) Comparison by age at treatment initiation: children aged ≥9 years showed higher progression compared to those <9 years (*p* = 0.04).

Figure 1 illustrates SE progression across adherence, concentration, and age subgroups.

### 3.4. Observed Versus Expected Axial Elongation (Model-Based Benchmark)

In the subgroup of 36 children with available AL measurements at baseline and 12 months, the mean observed axial elongation under low-dose atropine was 0.08 ± 0.23 mm/year.

Using the age- and race-adjusted meta-regression model by Brennan et al. [19], the expected untreated axial elongation for this cohort was 0.36 ± 0.09 mm/year.

The difference between observed and expected elongation was −0.28 ± 0.24 mm/year (95% CI: −0.36 to −0.20), indicating a substantial reduction in axial growth associated with atropine treatment (t(35) = −7.09, *p* < 0.001).

This corresponds to a relative reduction of approximately 78% in axial elongation compared with model-based expectations. The effect size was large (Cohen’s d = −1.18).

The observed reduction in axial elongation remained consistent across most participants included in the AL subgroup analysis, despite the variability expected in retrospective clinical cohorts. Although a small number of children demonstrated greater elongation rates, the overall distribution of outcomes was markedly shifted toward lower axial growth compared with the model-based untreated expectations. This finding is clinically relevant because even modest reductions in axial elongation during childhood may substantially reduce the cumulative lifetime risk of myopia-associated retinal complications, including myopic maculopathy and retinal detachment.

While direct comparisons between studies should be interpreted cautiously because of differences in ethnicity, baseline refractive status, follow-up duration, and treatment protocols, the elongation rates observed in the present cohort are broadly consistent with those reported in previous atropine clinical trials and European observational studies. The agreement between the observed treatment effect and previously published data supports the validity of the model-based benchmarking approach used in the present analysis and reinforces the potential utility of epidemiological reference models for contextualizing treatment efficacy in retrospective real-world research settings.

### 3.5. Predictors of Myopia Progression

The multiple linear regression model for SE progression was statistically significant (r^2^ = 0.164, *p* = 0.047). Age (β = −0.312, *p* = 0.008) and baseline SE (β = −0.257, *p* = 0.027) were the only significant predictors, indicating that older children and those with more negative initial SE values experienced greater myopic progression. No significant effects were observed for sex, adherence, family history, or atropine concentration.

The regression model for AL progression was not statistically significant (r^2^ = 0.144, *p* = 0.694). None of the predictors reached significance, although baseline AL showed a non-significant trend (β = −0.315, *p* = 0.121), suggesting that eyes with greater initial AL may elongate more slowly.

## 4. Discussion

This real-world study provides additional evidence supporting the effectiveness of low-dose atropine in reducing myopia progression in European children treated under routine clinical conditions. The mean SE progression (−0.08 D/year) and axial elongation (0.08 mm/year) observed in our cohort are substantially lower than those typically reported in untreated pediatric populations and closely align with the outcomes of controlled trials such as LAMP [15] and ATOM [9,11]. These findings reinforce the clinical value of low-dose atropine as a first-line intervention for myopia control.

From a clinical perspective, the low levels of refractive progression and axial elongation observed in this cohort support the growing integration of atropine therapy into routine pediatric ophthalmic and optometric care. Treatment outcomes in routine clinical practice are often influenced by factors that are difficult to replicate in randomized clinical trials, including variable adherence, differing follow-up schedules, parental preferences, and individualized prescribing strategies. The present findings suggest that meaningful reductions in myopia progression can still be achieved under these less controlled conditions, reinforcing the practical applicability of low-dose atropine outside highly standardized research environments.

A key contribution of this study is the integration of a model-based benchmark to contextualize treatment effects. Using the age- and race-adjusted meta-regression model proposed by Brennan et al. [19], we estimated that children in our cohort would be expected to elongate approximately 0.36 mm/year in the absence of treatment. In contrast, the observed elongation under atropine was only 0.08 mm/year, representing a 78% reduction relative to expected untreated progression. This magnitude of effect is comparable to, and in some cases exceeds, that reported in randomized clinical trials, despite the inherent variability of real-world practice. The large effect size (Cohen’s d = −1.18) further underscores the clinical relevance of atropine in slowing axial growth.

The use of an epidemiological model to estimate expected progression addresses a major challenge in contemporary myopia research: the ethical and practical limitations of including untreated control groups. Given the well-established efficacy of atropine, withholding treatment from progressing myopic children is increasingly difficult to justify [20]. Model-based benchmarks, therefore, offer a valuable alternative for interpreting treatment outcomes in real-world settings, particularly in retrospective designs where control groups are not feasible [21].

These model-based approaches may become increasingly relevant in future European myopia-control research, where placebo-controlled atropine studies remain comparatively limited and prescribing practices continue to vary across clinical settings. Although such methodologies cannot fully substitute for contemporaneous untreated controls, they may improve the interpretability and translational relevance of retrospective clinical studies by providing clinically meaningful reference estimates for expected untreated progression. When combined with standardized axial length monitoring and harmonized outcome assessment, these approaches may help facilitate more consistent interpretation of treatment outcomes across diverse clinical cohorts and support the continued integration of real-world evidence into evidence-based myopia management.

Beyond clinical outcomes, recent physiological evidence helps contextualize the dose–response patterns observed in atropine treatment. Ostrin and colleagues demonstrated that even very low concentrations of atropine (0.01–0.1%) induce measurable, dose-dependent changes in pupil dynamics and accommodative behavior lasting up to 24 h [22]. These findings provide a mechanistic framework for understanding why higher concentrations may offer increased efficacy but also carry a greater risk of visual side effects, such as reduced accommodation speed or increased pupil size. Although these functional parameters were not assessed in our pediatric cohort, they highlight the importance of balancing potency and tolerability in real-world prescribing, where subjective visual comfort may influence adherence. Future pediatric studies integrating functional metrics could help refine individualized dosing strategies to optimize both efficacy and visual performance.

Subgroup analyses revealed no significant differences in treatment response according to atropine concentration, adherence, sex, or family history of myopia. Although a dose–response trend was observed, with 0.05% showing the lowest SE progression, the differences were not statistically significant, likely due to sample size limitations. These findings are consistent with previous European studies reporting modest differences between low concentrations in real-world use [23,24]. The absence of adherence-related differences may reflect the relatively high adherence reported in our cohort or limitations in the subjective assessment of compliance.

Age at treatment initiation emerged as an important predictor of SE progression, with children aged ≥9 years showing greater myopic progression than younger children. This aligns with evidence suggesting that atropine may be more effective when initiated earlier, when axial growth is more rapid and potentially more modifiable [14]. Baseline SE also predicted progression, with more myopic children experiencing greater change, consistent with known risk profiles for faster progression.

The lack of significant predictors for axial elongation in the regression model should be interpreted cautiously. Axial length data were only available for 36 of the 76 participants, which reduced the statistical power of AL-related analyses and may have introduced selection bias. In addition, the relatively low variability in axial elongation observed under treatment may have further limited the ability to detect significant associations. Consequently, the absence of significant predictors does not necessarily indicate a true lack of relationship between the evaluated variables and AL progression.

Additionally, atropine concentration was determined clinically rather than through randomized allocation, reflecting routine clinical practice. As a result, treatment selection bias may have been introduced, particularly if higher concentrations were preferentially prescribed to children perceived to be at greater risk of progression. Therefore, comparisons between atropine concentrations should not be interpreted as evidence of equivalent efficacy across doses.

Future research should focus on prospective multicenter European cohorts with standardized axial length acquisition protocols, objective adherence monitoring, and systematic assessment of treatment tolerability. The incorporation of functional visual outcomes, including accommodative performance, photophobia, pupil dynamics, and quality-of-life metrics, may further improve understanding of the balance between efficacy and visual comfort across atropine concentrations. In addition, the integration of epidemiological growth models with individualized clinical data may help support more personalized approaches to myopia management, including risk-stratified treatment selection and longitudinal prediction of progression trajectories. Such approaches may become increasingly important as myopia-control strategies continue to evolve toward precision-based clinical care.

Other limitations of this study include its retrospective design, the absence of a contemporaneous untreated control group, retrospective adherence assessment based on parental reports and clinical documentation, and incomplete AL data for some participants. Because adherence was not evaluated using objective monitoring methods, compliance estimates may have been affected by recall bias and social desirability bias, limiting the precision of adherence-related analyses. Although the use of an age- and race-adjusted epidemiological model provides a useful benchmark for contextualizing axial elongation, it should not be interpreted as a substitute for a true untreated control cohort. The Brennan meta-regression was derived from heterogeneous untreated populations and may not fully capture the environmental, behavioral, clinical, or demographic characteristics of this European pediatric cohort. Therefore, the expected elongation values should be interpreted as population-level epidemiological estimates rather than individualized predictions or direct control-group outcomes.

Despite these limitations, this study provides meaningful real-world evidence supporting the effectiveness of low-dose atropine in European pediatric populations. By combining clinical outcomes with model-based expectations, it offers a nuanced and clinically relevant perspective on treatment efficacy under routine care conditions.

## 5. Conclusions

Low-dose atropine demonstrated real-world effectiveness in slowing myopia progression in a European pediatric population. After one year of treatment, both SE progression and axial elongation were markedly lower than values typically reported in untreated children. When contextualized using an age- and race-adjusted epidemiological model, observed axial elongation was reduced by approximately 78% relative to expected untreated progression, highlighting the substantial impact of atropine under routine clinical conditions.

Age at treatment initiation and baseline refractive error emerged as significant predictors of myopic progression, underscoring the importance of early identification and timely intervention. No statistically significant differences were observed across atropine concentrations, adherence levels, or demographic factors within the limitations of the present sample size.

By integrating clinical outcomes with model-based expectations, this study provides a nuanced and clinically relevant perspective on atropine efficacy in everyday practice. These findings support the continued use of low-dose atropine as a first-line therapy for myopia control in European children and emphasize the value of real-world evidence in guiding clinical decision-making.

## Figures and Tables

**Table 1:** Baseline characteristics of the treatment group.

Variable	Treatment Group (*n* = 76)
Mean Age (years)	8.7 ± 1.5
Sex (Male %)	43.4%
Initial SE (D)	−2.52 ± 0.80
Initial AL (mm)	24.42 ± 0.99
Family History	63.2%

SE: Spherical Equivalent; AL: Axial Length.

**Table 2:** Changes in SE and AL after 1 year of treatment with atropine.

Variables	Visit	*n*	Mean	SD	Min	Max
SE Progression (D)	12 M	76	−0.08	0.11	−0.50	0.25
AL Progression (mm)	12 M	36	0.08	0.23	−0.01	1.00

SE: Spherical Equivalent; AL: Axial Length.

## Data Availability

The datasets generated and/or analysed during the current study are available from the corresponding author on reasonable request.
